# Tidal modulation of the seismic activity related to the 2021 La Palma volcanic eruption

**DOI:** 10.1038/s41598-023-33691-1

**Published:** 2023-04-20

**Authors:** Luis Miguelsanz, José Fernández, Juan F. Prieto, Kristy F. Tiampo

**Affiliations:** 1 Institute of Geosciences (IGEO), CSIC-UCM, C/Dr. Severo Ochoa, 7., 28040 Madrid, Spain; 2 E.T.S. de Ingenieros en Topografía, Geodesia y Cartografía, Universidad Politécnica de Madrid, 28031 Madrid, Spain; 3 Cooperative Institute for Research in Environmental Sciences (CIRES), University of Colorado Boulder, Boulder, CO USA

**Keywords:** Natural hazards, Solid Earth sciences

## Abstract

The volcanic eruption at La Palma started on September 19, 2021. The eruption was preceded by a seismic swarm that began on September 11, although anomalous seismicity has been observed on the island since 2017. During the co-eruptive phase of the seismic activity, hypocenters depth was generally less than 15 km, save for the period between November 10 and November 27, when hypocenters ranged in the depth from 15 to 40 km. The eruption ended on December 13, 2021. We compute tidal stress for each earthquake at the hypocenter depth and find statistically significant correlations between the occurrence times of the earthquakes and the confining tidal stress values and stress rates. The correlation is depth-dependent, and ocean-loading tides have a stronger effect than body tides. We find that tidal stress variations contribute to the eruption onset and that certain explosive events, typical in Strombolian type volcanoes, seem to occur preferentially when the tidal stress rate is high. Our analysis supports the hypothesis that tides may modulate earthquake activity in volcanic areas, specifically during magma migration at shallow depths. A conceptual model is proposed, which could have a general application in the Canary Islands archipelago and other volcanic islands.

## Introduction

The cyclic tidal stress values due to lunar and solar tides range^1^ from 10 to 100 hPa. These values are small compared to earthquake stress drops^2^, which range from 1000 to 10^5^ hPa. However, tidal stressing rates may reach up to 10 hPa/hour, and therefore are often higher than tectonic stress rates between earthquakes^3^ (around 0.2 hPa/hour). Superposition of the fast-tidal stresses on the slow tectonic stresses could result in an earthquake triggering effect. For this reason, many researchers have studied the potential for significant correlations between tides and earthquakes, hoping that this knowledge can help improve seismic hazard assessment in warning systems. A detailed review can be found in Miguelsanz et al.^4^. Correlation between tides and volcano seismicity is a particularly interesting case, because changes in volcanic seismicity may be related to magma movements at shallow depths^5^. Significant correlations between tides and volcanic earthquakes have been found by several researchers^4–11^.

Seismic activity during volcanic unrest in El Hierro island, Canaries, 2011 and 2013, was the object of a study^4^. Statistically significant correlations between tides and volcanic earthquakes were found during specific phases of the volcanic unrest. Tidal stress values and rates were correlated with earthquake occurrence times, with vertical and E–W tidal components being the most significant^4^. They also showed that the influence of ocean-loading tides was stronger in El Hierro than that of solid Earth tides, and that tidal modulation was more significant in earthquakes whose hypocentres ranged in depth between 0 and 15 km than in those at greater depths. They concluded that tidal stress and tidal stress rates had an influence on overpressure and flow rates in the conduits and the shallow magma reservoirs, implying that the observed tidal modulation may be related to overpressure during magma migration.

La Palma, the second youngest island in the Canary Islands archipelago, underwent a volcanic eruption starting on September 19, 2021. This eruption was preceded by a seismic swarm^12^ that started on September 11. However, anomalous seismic activity on the island began in 2017, related to the existing volcanic unrest^12–15^.

The main purpose of this work is to study the existence of correlations between seismicity and tides in the context of the volcanic eruption in La Palma, 2021, and investigate its evolution since 2017, when the first anomalous seismic activity was recorded. We compare the correlation patterns at La Palma unrest to those obtained for the El Hierro volcanic unrest^4^.

## La Palma 2021 volcanic eruption

**Figure 1:**
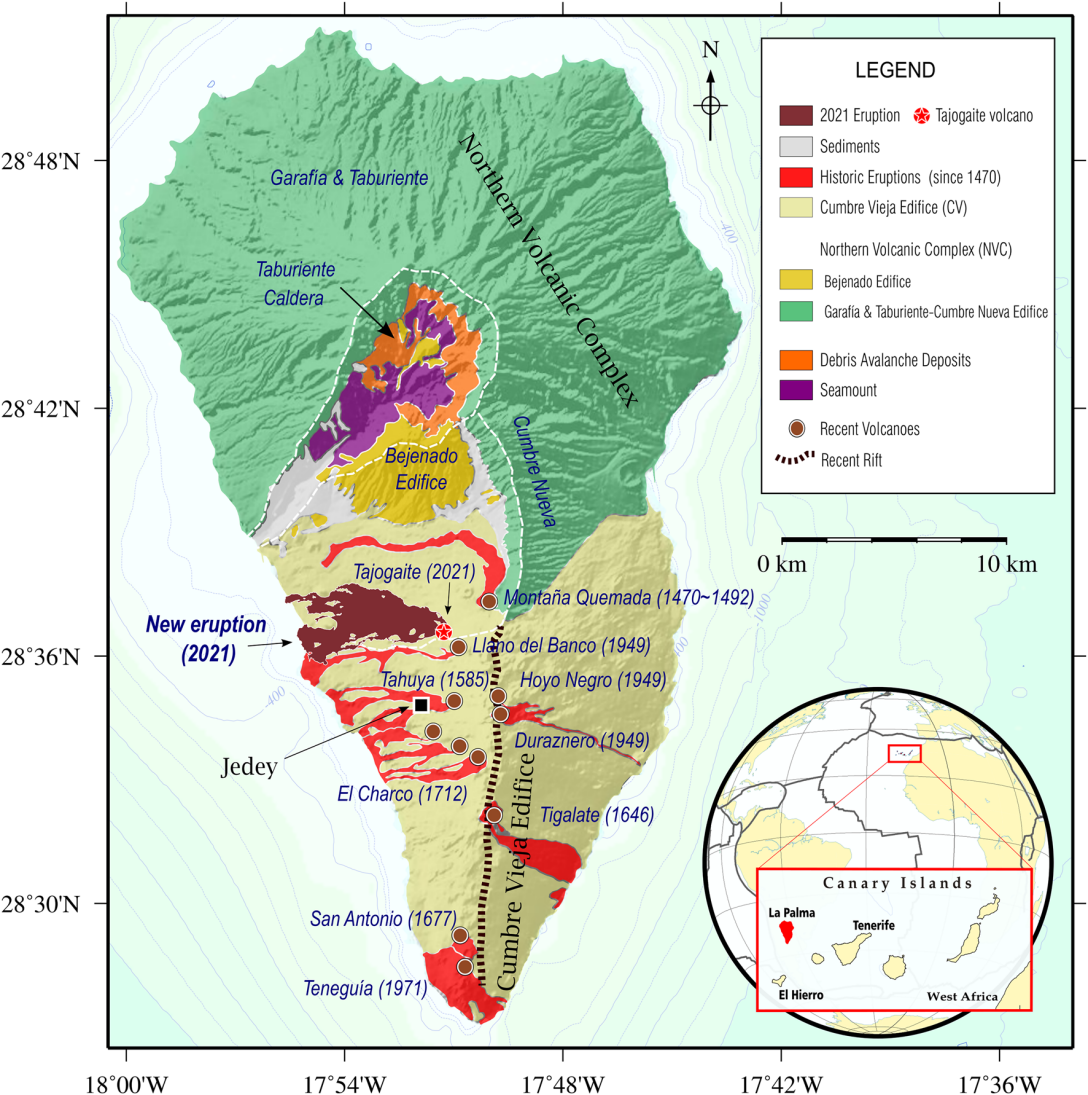
Geographical location and geological and volcanological elements of La Palma island. Geologic elements of La Palma showing the main volcanic complexes, ridges as well as the last historical eruptions on the Island. Upper insets show the scale and keys description. The lower inset shows the location of Canary Islands and La Palma island. GMT software (www.generic-maping-tools.org) was used to create this figure.

The island of La Palma primarily is composed of two large volcanic structures (Fig. 1): the Northern Volcanic Complex (NVC) and Cumbre Vieja (CV). NVC hosts the volcanic buildings of Garafía, Taburiente, Cumbre Nueva and Bejenado, whereas the CV edifice is a younger rift-type structure, along which both recent and historical on-land volcanic activity has taken place. Magma arrives to shallow crustal regions below the island from depth, probably following pre-existing intrusion paths in a zone with low density and weakness^15–17^ below the Jedey zone. Seismicity that occurred in 2017–2020 appears to be related to rock fracturing below CV^14^, opening the path towards the shallow and short-term reservoir below the Jedey zone^17^ (Fig. 1). The magmatic recharge of this shallow reservoir explains the seismic swarms of early and mid-2021. The seismic swarm that started on September 11, 2021, preceded and accompanied the La Palma 2021 eruption onset.

The eruption, which produced the Tajogaite volcano, began at 14:10 UTC on 19 September in Cabeza de Vaca (El Paso municipality), after an M = 3.8 earthquake occurred at 10:16 UTC, at 2 km depth^12^. Large explosions were heard, and a gas-and-ash plume as high as 5 km above sea level (a.s.l.) was observed. The seismic network detected a volcanic tremor signal which exhibited strong variations in amplitude over time. A cone formed the following day^18^ and eruptive vents opened, collapsed, and closed, grouping around a ~700 m long fissure trending NW-SE^17,19^. In the following weeks, intense effusive and explosive activity occurred with phreatomagmatic pulses, the expansion of the lava flows, and daily ash emissions^12,20^.

In the first stage or the eruption, most of the hypocentres were less than 15 km depth, although a significant percentage of hypocentres are in the focal depth range 25–40 km. At 14:00 UTC on 24 September, volcanic tremor reached its highest value since the beginning of the eruption. Two new vents opened shortly after, with lava running downslope and covering previous flows. This increase in the amplitude of the volcanic tremor is coincident with the greater intensity of the Strombolian explosions in the eruptive vents. Episodes of explosive activity followed one after the other during the following weeks. In the main cone, explosions and lava overflow occurred at 19:00 UTC on 22 October. A partial collapse of the main cone occurred on 23 October, throwing large blocks to a secondary cone, and causing lava spill. Strong explosions were felt at 11:30 UTC, and the explosive activity continued over the next days, with two partial collapses of the main cone on 25 October, around 16:00 UTC and 20:00 UTC, and another on 26 October. On 29 October, intense explosions were heard over several hours, starting around 11:00 UTC. These explosions produced a great amount of ashfall that descended mainly over the west and northwest sections of the island. Seismicity remained high, with three events of magnitude *M* = 5 between 30 October and 3 November, and several earthquakes greater than *M =* 4. A decrease in volcanic tremor was observed on 2 November, and tremor levels remained low during the following weeks, with only occasional increases^21–30^.

As of November 10, hypocentre depth changed, with most occurring at depths of 15–40 km. A seismic swarm was detected on 11 November after another *M* = 5 earthquake al 3:37 UTC, approximately the same time as the tremor signal briefly intensified^12^. Seismicity remained high. The daily number of earthquakes reached a maximum of 230 events on 17-18 November. An *M* = 5.1 earthquake, the largest event to that date, was detected on 19 November^28,29^.

The trend change in hypocentre depth had reversed by November 27, with deep events diminishing while the seismic activity intensified at mid-level depths of 10–15 km. At approximately 2:00 UTC on 28 November, new vents opened on the main cone. Seismicity remained high, and lava continued moving westward, encompassing a flow field composed of up to twelve overlapping lava flows and two lava deltas. Strombolian activity in the main cone was particularly intense between 1 and 3 December, with a new fissure opening on the SE flank. Several lava flows from different eruptive vents continued travelling in the west and southwest directions^30,31^.

By 8 December, eruptive activity in the main cone was low, save for some Strombolian type episodes. Seismicity also was low, particularly at depth ranges of 30–40 km. On 12 December, strong Strombolian activity at 11:00 UTC and 16:30 UTC was coincident with high tremor values. Ash emissions reached a height of 6 km, and bombs were ejected hundreds of meters away. The eruption ended on December 13. That day, there was important explosive activity between 16:45 and 18:00 UTC, along with ejection of bombs^32^ and gas-and-ash plumes that reached an altitude^33^ of 8500 metres asl. After that, tremor started to decrease and by 22:21 UTC it had reached levels similar to those prior to the volcanic eruption^12^. No lava flows were observed over the following days, tremor remained at background levels, and seismicity remained low^34^.

**Figure 2:**
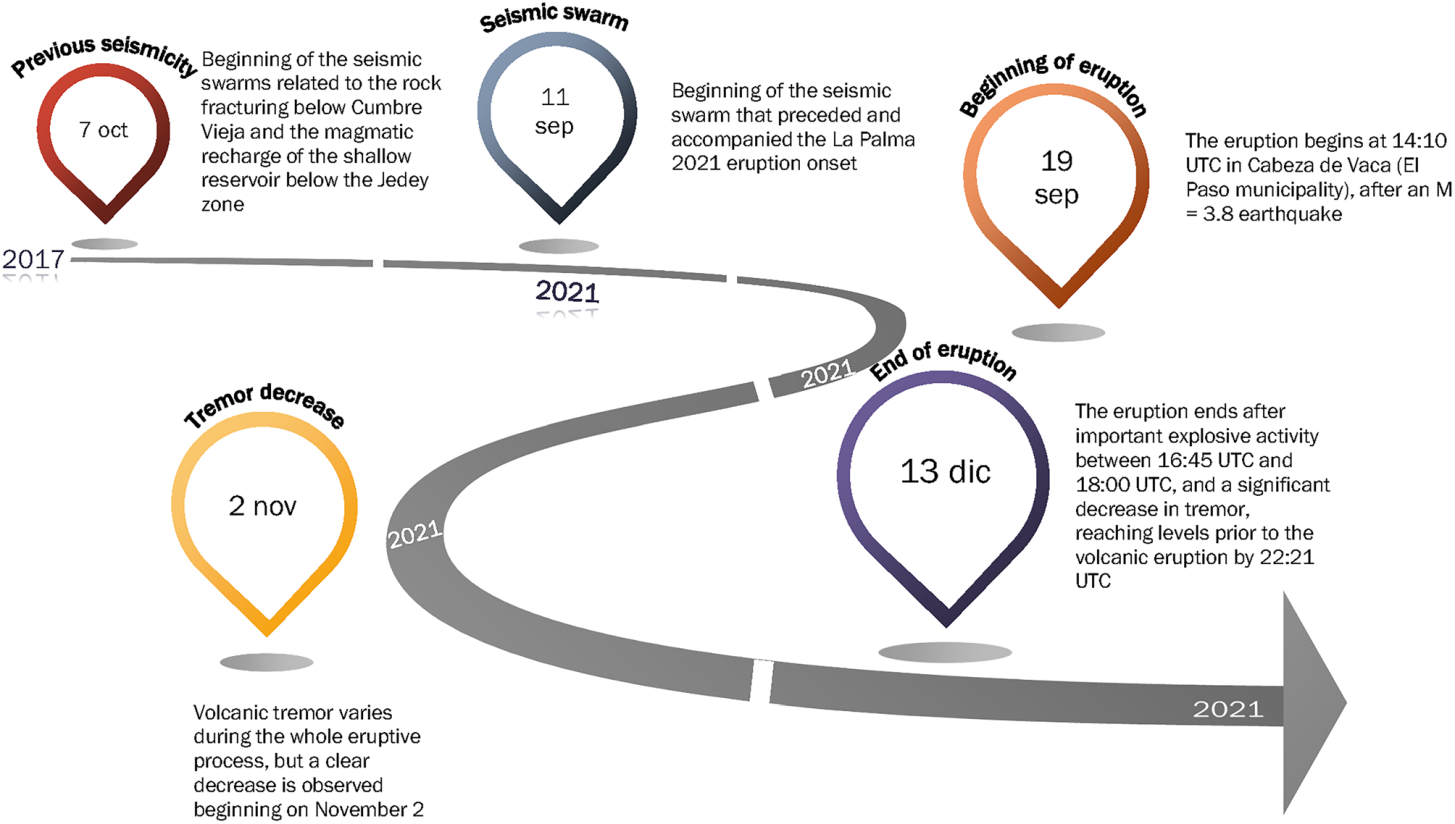
Evolution of the volcanic unrest in La Palma. The figure shows, in schematic form, the evolution of the volcanic unrest in La Palma since the earthquake swarms beginning in 2017 until the end of the volcanic eruption on December 13, 2021.

The altitude of the volcanic cone built during the eruption is 1131 m, asl approximately 200 m over the pre-eruptive topography, with an estimated volume of emitted lava^18,35^ of about 217 Mm^3^. The greatest vertical deformation was 33 cm, recorded on 24 October^12^. The lava flows buried more than 2800 buildings and nearly 1000 hectares. Half of the historical volcanic eruptions in the Canary Islands have taken place in the CV rift zone, suggesting that this rift zone is statistically the most likely setting of future eruptions in the archipelago area^33^. Figure 2 shows the timeline of the evolution of the volcanic unrest.

## Earthquake data

Earthquake data for the period 2017/10/07–2021/12/31 was obtained from *Instituto Geográfico Nacional*^36^. We considered different time periods (phases) in our study. As the location of small earthquakes may have significant errors^37^ and we wanted to avoid biased data as far as possible, we removed all earthquakes with magnitude less than the magnitude of completeness *M*_*c*_ in the different phases considered.

*Phase 0* comprises all seismicity from 2017/10/07 to 2021/09/10. The seismicity belonging to this phase is largely composed by a series of swarms of very low magnitude (*M* < 3). Most earthquakes occurred at a depth greater than 25 km, including events with focal depth between 30 and 40 km. The minimum magnitude of completeness *M*_*c*_ of the catalog corresponding to *Phase 0*, calculated according to the maximum curvature technique^38^, varies greatly throughout the years comprising this period (see Supplementary Fig. 1), as the seismic activity changes from one year to another, with few earthquakes recorded between 2018 and 2019. The use of the MAXC technique applied to the *Phase 0* catalog as a whole provides a value *M*_*c*_ = 1.6, but we will follow here *Mignan and Woessner*^38^, assigning to the *Phase 0* period the value *M*_*c*_ = 1.9, which is the maximum *M*_*c*_ calculated for the different subperiods considered (namely, it is the value obtained for the year 2018). It must be noted that earthquakes in *Phase 0* show significant hourly variations, as 68% of the events occur during nighttime (between 18:00 and 6:00). Anthropic noise may be a reason, but also swarming of the events. Declustering the *Phase 0* catalog with the Reasenberg algorithm^39^ reduces the day/night bias to 61%, so henceforth we will use this declustered version in the analysis of *Phase 0*.

*Phase 1* [2021/09/11–2021/09/19] includes seismicity immediately prior to the volcanic eruption beginning on September 19. The seismic swarm which began on September 11 contains a greater number of events than any of the other swarms that preceded the eruption, including several earthquakes of magnitude *M* > 3. Focal depth is less than 11 km for almost all events in this stage. The magnitude of completeness *M*_*c*_ is 1.9 for the *Phase 1* catalog (see Supplementary Fig. 2). The earthquakes in *Phase 1* shows also a clear day/night bias, as 62% of the events occur during nightly hours. Anthropogenic noise should not be discarded, but the fact that all the seismicity in this *Phase 1* is mainly a swarm may be the reason. Declustering is not an option here, as the 811 earthquakes in this *phase* would then be reduced to 15 events. Nevertheless, Supplementary Table 1 shows that *M*_*c*_ = 1.9 is the magnitude cutoff with the lowest ratio (night events)/(total number of events).

Finally, *Phase 2*, 2021/09/20–2021/12/13, includes the seismicity coincident with the eruption. During this period, there were several events of *M* ≥ 5. Focal depth is between 10 and 15 km for most events, although approximately 15% of the earthquakes occur at greater depths, including some events with hypocentres around 40 km depth. The magnitude of completeness of the whole period is *M*_*c*_ = 2.6, a value higher than the one obtained in both *Phase 0* and *Phase 1*, probably because of the tremor related to the volcanic activity. No significant asymmetry in day/night is observed in this catalog, suggesting that the seismic network operates with a better detection capability in this *Phase 2* than in the two previous *phases*, or that the volcanic tremor overwhelms the anthropogenic noise. A review to the list of seismic stations in La Palma (see Supplementary Table 2) shows that a new station was added to the network soon after the beginning of the eruption, on September 21. The tremor varies during the eruptive process, with a clear decrease starting as of November 2^26–32^. We split *Phase 2* in two periods *P1* [2021/09/20–2021/11/01] and *P2* [2021/11/02–2021/12/13] and obtain *M*_*c*_ = 2.8 for *P1* and *M*_*c*_ = 2.6 for *P2* (see Supplementary Fig. 3).

## Results

We study the existence of correlations between tidal confining stress and the occurrence of the earthquakes for the three phases. See “Methods” section for a description of the Schuster’s test used and a discussion about its statistical significance. Table 1 shows the results which are described in the following sub-sections.

**Table 1:** Summary of the correlations found between tidal stress and origin time of the events.

Phase	Period	Number of events (*M* ≥ *M*_*c*_)	*P*_*s*_ value	$\overline{B }$
Phase 0	2017/10/07–2021/09/10	150	**<< 0.0001**	**337395.0170**
Phase 1	2021/09/11–2021/09/19	811	**0.0044**	15.2831
Phase 2	2021/09/20–2021/12/13	5668	0.3659	1.0000
Phase 2 – P1	2021/09/20–2021/11/01	2215	**0.0179**	5.1045
Phase 2 – P2	2021/11/02–2021/12/13	2595	0.1257	1.4109

*M*_*c*_ is the magnitude of completeness, which is different in every set or subset of events. See Section “Earthquake data”. *P*_*s*_ is the probability that the phase distribution to be random, according to Schuster’s test. $\overline{B }$ is the upper bound of the Bayes factor calculated as a function of the *p*-value *P*_*s*_, as told in the “Methods” section. Occurrences where *P*_*s*_ < 0.05 or $\overline{B }$ > 16 are in bold. Note that *Phase 2* has been divided in two periods: *P1* [2021/09/20–2021/11/01] and *P2* [2021/11/02–2021/12/13].

### Phase 0 [2017/10/07–2021/09/10]

According to the values of *P*_*s*_ and $\overline{B }$ (Table 1), the correlation for *Phase 0* (declustered) is statistically significant. Supplementary Fig. 4 shows the histogram of tidal confining stress phase angles corresponding to this stage (see “Methods” for definition of tidal phase angle *ϕ*).

For the histograms used in this study, earthquakes are collected in phase ranges of 30° width. The sinusoidal curve applied to the frequency distribution is obtained by least squares fitting of the expression

1 $$F(\phi )={F}_{0}+{F}_{1}\mathit{cos}(\phi -\alpha ),$$

where *ϕ* is the phase angle, *F*_0_ is the mean frequency, *F*_1_ is the amplitude of the curve and α is the phase of the curve^1^. A triangle marks the peak of the fitted curve associated with the histogram, occurring on the phase angle maximizing Eq. (1). In this case, approximately 25 % of the events occur during the phase range 210° < *ϕ* ≤ 240°, and the fitted curve peaks around phase angle *ϕ* = 214°.

### Phase 1 [2021/09/11–2021/09/19]

This phase covers the seismic activity immediately prior to the beginning of the volcanic eruption on September 19, 2021. According to the Schuster test, the correlation between tidal confining stress and the origin time of the events is statistically significant, with *P*_*s*_ = 0.0044 (Table 1). The upper bound of the Bayes factor is $\overline{B }$= 15.28311, which is almost the significant threshold proposed by *Bayarri* et al.^40^ (see “Methods”). Supplementary Fig. 5 shows the histogram of tidal confining stress phase angles corresponding to *Phase 1*. The fitted curve peaks around phase angle *ϕ* = 119°.

### Phase 2 [2021/09/20–2021/12/13]

In this case, the correlation between tidal confining stress and the occurrence time of the earthquakes is not statistically significant (Table 1). Supplementary Fig. 6A shows the histogram of tidal confining stress phase angles corresponding to *Phase 2*, which is the stage coincident with the volcanic eruption. Nevertheless, if we divide *Phase 2* into two periods (Table 1), we find a statistically significant correlation for period *P1* [2021/09/20–2021/11/01], at least according to the Schuster test (Supplementary Fig. 6B). The fitted curve peaks around phase angle *ϕ* = 33°, quite close to the maximum tidal stress. The lack of a significant correlation in the subsequent period *P2* (Supplementary Fig. 6C) may be due to a change in magma plumbing conditions at CV volcano after the partial collapses and explosions on the main cone during the last days of October.

To assess the relevance of focal depth in the correlations, we divided the events in *Phase 2 – P1* into two subsets: *2A*, earthquakes with a focal depth less than 15 km; and *2B*, events with depth greater than 15 km. Histograms corresponding to both *2A* and *2B* are shown in Supplementary Fig. 7. Whereas the correlation in subset *2B* is not statistically significant (see Table 2), correlation found for subset *2A* (Table 2) is significant for both statistical tests, *P*_*s*_ and $\overline{B }$. The peak of the fitted curve is around *ϕ* = 39°, close to the maximum of tidal stress. Although the statistical significance of the tests may depend on the number of events considered, as it is easier to find correlations in small datasets^3^, it is clear that in this case depth is a key factor, and set *2A*, which is seven times greater than *2B*, is the one featuring the most statistically significant correlations.

**Table 2:** Summary of the correlations found for subsets *2A* and *2B*.

Subset	Depth range	Number of events	*P*_*s*_ value	$\overline{B }$
*2A*	< 15 km	1934	**0.0018**	**31.8873**
*2B*	> 15 km	281	0.2824	1.0303

*P*_*s*_ is the probability that the phase distribution to be random, according to Schuster’s test. $\overline{B }$ is the upper bound of the Bayes factor calculated as a function of the *p*-value *P*_*s*_, as told in the “Methods” section. Occurrences where *P*_*s*_ < 0.05 or $\overline{B }$ > 16 are in bold.

The results of a Monte Carlo permutation test^38,41^ applied to earthquakes in *Phase 0*, *Phase 1*, and *Phase 2 – subset 2A* (Table 3; see “Methods” for a description of the permutation test) support the statistical significance previously provided by the Schuster test and the upper bound $\overline{B }$ of the Bayes factor. This analysis is very important, due to the swarming of the events.

**Table 3:** Results of the application of Schuster’s test and a Monte Carlo permutation test (10,000 simulations) to *Phase 0*, *Phase 1* and *Phase2-subset 2A* (see “Methods” for a description of the permutation test).

Phase	Number of events	*P*_*s*_ value	Segment length: 1°		Segment length: 2.5°	
			NSIG (P*< *P*_*s*_) for 10,000 permutations	SL *MC*_*sl*_	NSIG (P* < *P*_*s*_) for 10,000 permutations	SL *MC*_*sl*_
Phase 0	150	**< 0.0001**	0	**0.0001**	0	**0.0001**
Phase 1	811	**0.0044**	37	**0.0038**	51	**0.0052**
Phase 2 – 2A	1934	**0.0018**	7	**0.0008**	12	**0.0013**

*P*_*s*_ is the probability that the phase angle distribution in the original catalogue to be random, according to Schuster’s test. For each permutation, a Schuster probability value P* is obtained. NSIG is the number of permutations where P*< *P*_*s*_ for each cluster. SL means “significance level”. *MC*_*sl*_ is the significance level of the permutation test, as explained in the “Methods” section. Different segment lengths were applied. Occurrences where *P*_*s*_ < 0.05 or *MC*_*sl*_ < 0.05 are in bold.

## Discussion

**Figure 3:**
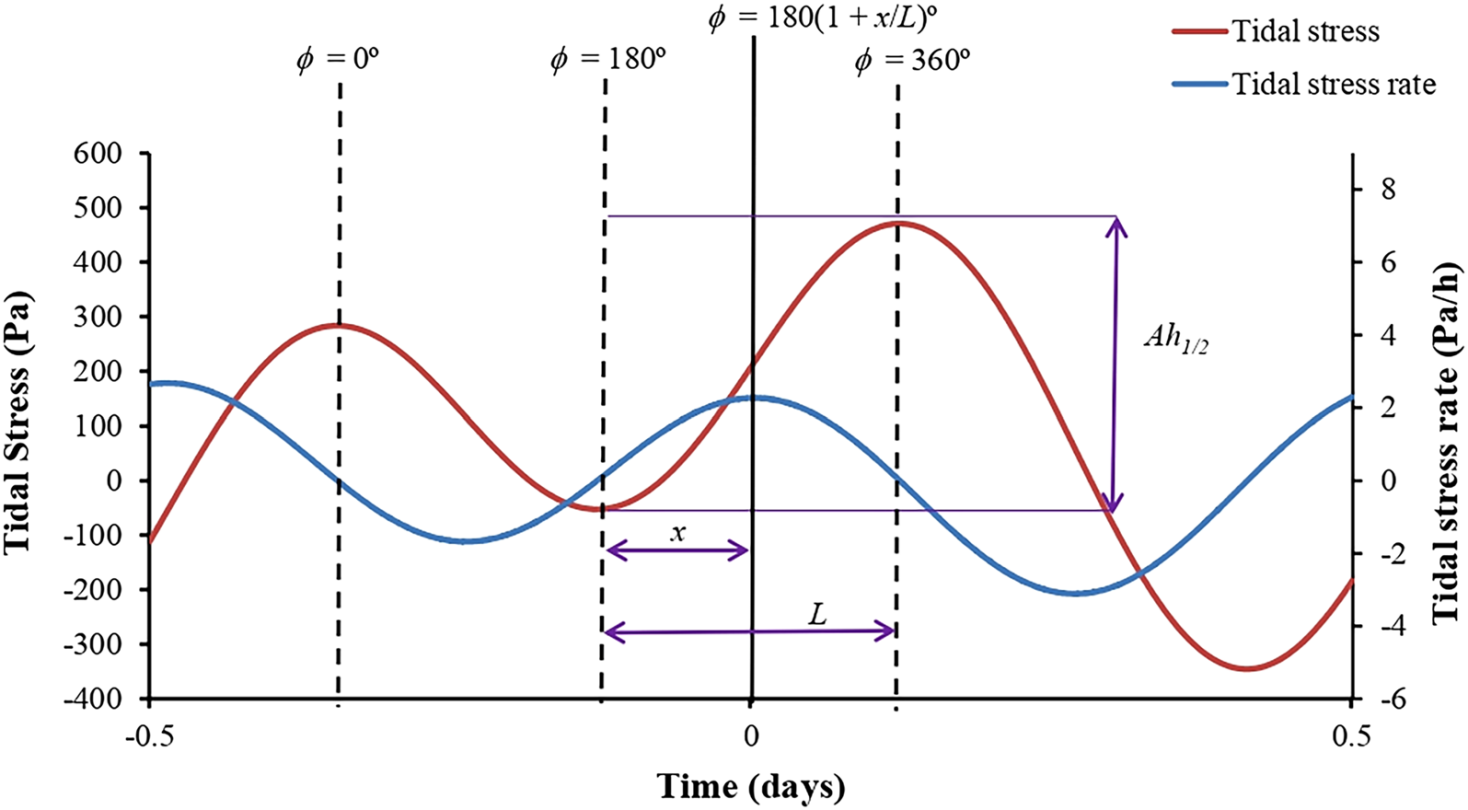
Schematic of the tidal phase method^48^. The terms *Ah*_*1/2*_ and *L* are the amplitude and time length of the half cycle in which the event occurs, respectively. *x* is the time difference between the occurrence time of the earthquake and phase *ϕ* = 180°, positive when the event occurs after phase *ϕ* = 180° and negative when it happens prior to phase *ϕ* = 180°. The tidal stress rate is shown as the blue line. Note that maximum increasing tidal stress rate occurs around tidal stress phase *ϕ* = 270°, whereas maximum decreasing tidal stress rate happens around tidal stress phase *ϕ* = 90°.

Previous research indicated that the strongest gradients of tidal variations might be more important for the modulation of seismicity than the absolute value of the tidal stress^4–6^. Therefore, stressing rates may be a major controlling factor, rather than the absolute maximum/minimum stress change values, to explain the correlation between tides and earthquake occurrence at La Palma. Figure 3 shows the difference between the graphs of tidal stress and tidal stress rate.

With that in mind, we calculated tidal confining stress rates corresponding to *Phases 0, 1,* and* 2* to determine if the events occur more frequently when tidal stress rate is higher. As in the previous section, we use the total tides (load tides plus body tides) in the calculation of the tidal phase angle *ϕ*. The results for the statistical tests are shown in Table 4, and the corresponding histograms appear in Supplementary Fig. 8.

**Table 4:** Summary of the correlations between tidal stress rate and origin time of the events.

Phase	Period	Number of events (*M* > *M*_*c*_)	*P*_*s*_ value	$\overline{B }$
Phase 0	2017/10/07–2021/09/10	150	**<< 0.0001**	**397253.62**
Phase 1	2021/09/11–2021/09/19	811	**0.0011**	**49.2967**
Phase 2	2021/09/20–2021/12/13	5668	0.3314	1.0051
Phase 2 – P1	2021/09/20–2021/11/01	2215	**0.0143**	6.0445
Phase 2 – P2	2021/11/02–2021/12/13	2595	0.1324	1.3741
Phase 2 – Set 2A	2021/09/20–2021/11/01	1934	**0.0013**	**41.4482**

*P*_*s*_ is the probability that the phase distribution to be random, according to Schuster’s test. $\overline{B }$ is the upper bound of the Bayes factor calculated as a function of the *p*-value *P*_*s*_, as told in the “Methods” section. Occurrences where *P*_*s*_ < 0.05 or $\overline{B }$ > 16 are in bold. As in Table 1, *Phase 2* has been divided in two periods: *P1* [2021/09/20–2021/11/01] and *P2* [2021/11/02–2021/12/13].

There are three sets of events featuring statistically significant correlations between tidal stress rate and origin time of the earthquakes according to both statistics *P*_*s*_ and $\overline{B }$: *Phase 0*, *Phase 1*, and *Phase 2 – Set 2A* (i.e. events with depth < 15 km)*.* In *Phase 0*, approximately 20 % of the events occur during the phase range 300º < *ϕ* < 330°, quite close to the maximum tidal stress rate (Supplementary Fig. 8A). On the contrary, earthquakes in *Phase 1* are more prone to occur when tidal stress rate is close to the minimum, as suggested by a fitting curve with peak around *ϕ* = 192° (Supplementary Fig. 8B). Note that, as we consider tidal stress positive in extension, minimum tidal stress is indeed maximum tidal stress in compression. Finally, events in *Phase 2 – Set 2A* seem to occur preferentially after maximum tidal stress rate was reached, with more than 11% of the events during the phase range 60° < *ϕ* < 90° and a fitting curve peaking around *ϕ* = 114° (Supplementary Fig. 8C).

**Figure 4:**
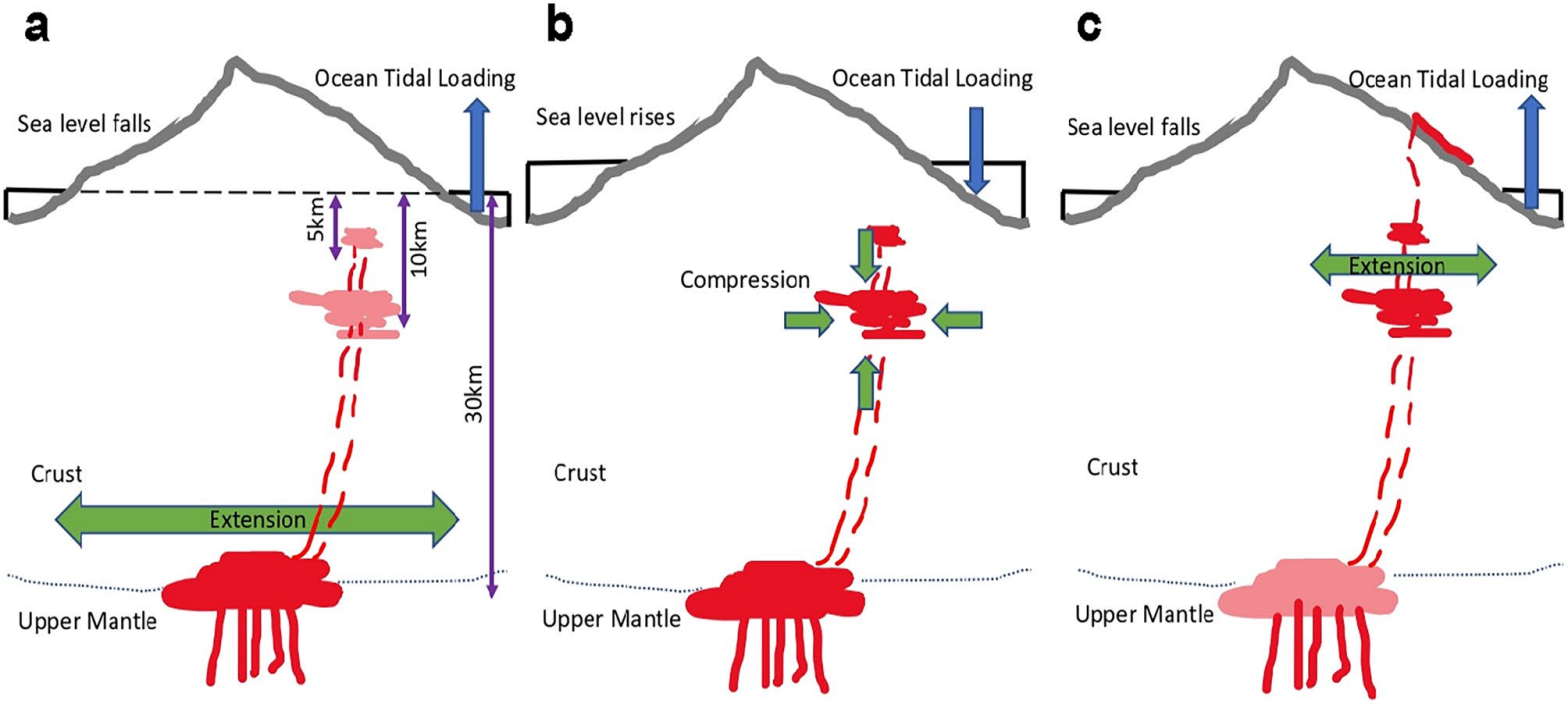
Schematics of the proposed sequence of magmatic activity related to the observed correlations between tides and earthquakes. Although extension and compression alternate on a daily basis, seismicity in *phases* 0 and 2 is favoured by extension, whereas in phase 1 compression is dominant. (**a**) Time period 2017-10-07 to 2021-09-10 (*Phase 0*). At low tides, sea level falls and ocean loading diminish, which means relative compression decreases and extension increases. High values of extensional tidal confining stress rates may facilitate a relative increase in magma upwards flow and encourage increased seismicity. The earthquakes occurred during this period opened new paths in the magma ascent (red dashed lines) from the deep reservoir at 25–30 km to a shallower one at 10 km, and to an even shallower reservoir around ~1–5 km depth, which filled shortly before the eruption. (**b**) Time period 2021-09-11 to 2021-09-19 (*Phase 1*). Lateral growth of the reservoir at 10 km depth has ended, and conditions in this reservoir are more sensitive to small changes in the compressional tidal confining stress rates, until the beginning of the eruption on September 19. At high tides, sea level rises and ocean loading increases, promoting magma pressure build-up. Tidal confining stress rate compression, together with magmatic stresses, would over-pressurize this reservoir, causing potential failure and withdrawal through zones of weakness to ascend and favour seismic activity. (**c**) Time period 2021-09-20 to 2021-11-01 (*Phase 2–Set 2A*). After the start of the eruption on September 19, 2021, pressure within the magma reservoirs at 10 and 2–5 km depth drops, and seismicity is promoted by maximum values of extensional tidal confining stress.

Figure 4 shows our interpretation of the volcano dynamics that explain the correlations. Although extension and compression alternate on a daily basis, seismicity in *phases* 0 and 2 is favoured by extension, whereas in phase 1 compression is dominant. In 2009–2010, magma coming from a deep reservoir at 25–30 km depth begins to accumulate in a shallower reservoir at 8–10 km depth^14^, expanding laterally. In the context of the deep reservoir, at low tide and the lowering of sea level, the weight of the ocean over the seafloor around the island reduces, resulting in a relative decrease in compression and an increase in extension (Fig. 4a).

Correlation between tidal stress rate and the origin times of the earthquakes in *Phase 0*, with hypocentres primarily at depths greater than 25 km, should be highest for high values of extensional tidal confining stress rates, explaining the histogram in Supplementary Fig. 8A.

Pressure sources initially located along the border separating the Northern Volcanic Complex and CV^14^ enhanced magma movement towards an even shallower reservoir below the Jedey zone (Fig. 1), in a low-density zone at ~1–5 km depth^17^.

The earthquakes occurred during 2017–2020 appear to be related to rock fracturing, opening a path^14,17^ towards the shallow depth and short-term reservoir below the Jedey area, whereas the seismic swarms of January and June 2021 may be related with the magma recharge of this shallow reservoir^17^. By the beginning of the 2021-09-11 to 2021-09-19 time period (*Phase 1*), lateral growth of the 10 km depth reservoir had finished, suggesting that conditions in this reservoir may be more sensitive to small changes in the compressional tidal confining stress rates, until the onset of the eruption on September 19 (Fig. 4b). With sea level rise at high tide, compression exerted on the seafloor by the body of water increases, promoting magma ascent and the build-up of pressure within the magma reservoir. Compressional tidal confining stress rate, together with tectonic and volcanic stresses, would encourage magma to migrate into zones of weakness to ascend, favouring seismic activity in the process. Note that this would explain the correlation between tidal stress rate and the origin times of the earthquakes in *Phase 1,* whose hypocentres are mostly in the depth range between 12 and 6 km, as seen in the histogram in Supplementary Fig. 8B. Finally, during the period between the beginning of the eruption and November, 1st (*Phase 2 – Set 2A*; Fig. 4c), pressure drops within the two shallow magma reservoirs^15–17^ between ~1–10 km depth and seismicity is promoted by maximum values of extensional tidal confining stress, as suggested by Supplementary Fig. 7A. Supplementary Fig. 8C agrees with this interpretation because of the relation between tidal stress and tidal stress rate (see Fig. 3), such that maximum increasing tidal stress occurs around tidal stress rate phase *ϕ* = 120º, not far from the peak of the fitted curve of the histogram in Supplementary Fig. 8C, which is around *ϕ* = 114º. No statistically significant correlation was found during period *P2* [2021/11/01 - 2021/12/13] (Table 4). This may be explained by changes in the magma dynamics following partial collapse and explosions on the main cone at the end of October. In addition, possible readjustment of the magmatic plumbing system may be associated with the final phase of the eruption, when there is no significant shallow storage^17^ and magma from the cones originates primarily from deep sources.

Supplementary Fig. 9 shows the comparison between tidal volume strain calculated for an imaginary focus whose epicenter is at the geographical center of all the epicenters and whose depth is the mean depth of events in the catalogue and the number of earthquakes at 2-hour intervals during the period 2021/08/31–2021/12/15. In this graph we use only earthquakes with magnitude greater than or equal to 2.8, which is the greatest magnitude of completeness in the periods or sub-periods considered. Most events in the earthquake swarm prior to the eruption occur when the tidal strain amplitudes are low, but the beginning of the eruption, and some coincident seismic activity, occurs when the amplitudes are increasing towards the maximum during that fortnight. Therefore, it appears that tidal stress variations contribute to the eruption onset.

Supplementary Fig. 10 shows the three components of tidal strain (N–S, E–W, and vertical) for the same period as Supplementary Fig. 9. The most important tidal component is E–W tidal strain. A similar plot featuring the three components of tidal stress (Supplementary Fig. 11) shows that the amplitudes in the E–W component are greater than the amplitudes in the other two. A second graph covering the same time period (Supplementary Fig. 12) shows the tidal stress decomposed into body tides and ocean tides. An analysis of half cycle amplitudes (see definition in Fig. 3) induced separately by ocean tidal loading stress and solid earth tidal stress reveals that tidal loading stress is, on average, six times greater than tidal body stress (see data in the repository Zenodo.org), a ratio similar to that found in El Hierro^4^.

We also studied the correlation between the occurrence time of the earthquakes and E–W and N–S tidal tilt components. Histograms are shown in Supplementary Figs. 13 and 14, respectively. Correlations are overall weaker than the ones found for tidal stress or tidal stress rates (Tables 1 and 4; Supplementary Tables 3 and 4), particularly during time period *Phase 2 – P1*. Nonetheless, correlations are stronger for the N–S tilt component than for the E–W component, potentially because tidal amplitudes are greater in the N–S tilt component, as shown in Supplementary Fig. 15.

In summary, we show that the correlation between seismicity and tides evolves throughout the different phases of the unrest and eruption, from the first anomalous seismicity in 2017 to the end of the eruption in December 2021, applying various statistical techniques. In particular, we found that tidal stress variations contribute to the eruption onset.

Tides modulated the seismic activity not only during the volcanic eruption, but also the seismic swarms that have occurred since 2017, associated with the volcanic unrest. We found that the confining tidal stress values and their rates show statistically significant correlations with the times of occurrence of the earthquakes. We determine that ocean-loading tides are stronger than body tides, as in the case of El Hierro island^4^.

In addition, although tidal modulation in the first of the stages of the seismic activity (*Phase 0*) largely affects deep seismicity (around 30 km depth), the correlation seems to be limited to depths lesser than 15 km in the following stages. We have shown that the E-W tidal stress component has greater amplitudes than both the N–S and vertical tidal stress components, whereas it is the N–S component which is more relevant for tilt tides. For the El Hierro unrest, E–W and vertical tidal stress components had similar magnitude for the equivalent phase^4^.

Our results suggest that, during the first stage, high values of extensional tidal confining stress rates enhanced both seismicity and upward migration of magma towards the shallower reservoirs. Then, compressional tidal confining stress rates helped in promoting the increase of magma pressure in the 10 km depth reservoir until failure and magma ascended to the shallow reservoir in zones of weakness, promoting seismicity. Finally, after the beginning of the volcanic eruption, high values of tidal confining stress modulated the seismicity coincident with the eruption until a series of collapses of the main cone during the last days of October 2021 modified the magma plumbing conditions at the volcano and the correlation disappeared.

There are similarities between the results found in this research and the correlations between tides and earthquakes during the El Hierro 2011–2013 volcanic unrest^4^. In both cases, ocean-loading tides are stronger than solid Earth tides and correlation occurs mainly at a hypocentre depth range between 0 and 15 km, although in the case of La Palma tidal correlation in the first stage (*Phase 0*) occurs for earthquakes with depths greater than 25 km. The magmatic activity related to the observed correlations also is similar for the two situations. In both cases, there is a first stage in which extensional tides facilitate an increase in magma upwards flow towards shallower reservoirs, followed by a second stage (the most immediate time period previous to the eruption) where compressional tides promote magma pressure build-up in the shallower reservoirs. There are differences in the following stage. Whereas in the case of El Hierro, there was not a significant correlation between tides and earthquakes during the co-eruption phase, in the case of La Palma that correlation exists, although it vanishes after October 2021, around the middle of the co-eruption phase. The explanation for that lack of correlation in El Hierro^4^ could be that seismicity during that co-eruption phase may not be due to migration of over-pressurized magma, but to the gravitational compaction of the magma plumbing system due to magma withdrawal^42^, or that the fast-rising magma during the eruption produced stronger stress changes than the ones due to tidal stress. The same processes may explain the lack of correlation in La Palma during *Phase 2 – P2*, with the series of collapses of the main cone during the last days of October, potentially modifying the magma plumbing conditions at Tajogaite volcano in CV. Also, the eruption of El Hierro was submarine while that of La Palma was subaerial, which also may have affected in the correlations.

Our analysis agrees with previous studies supporting the hypothesis that tides may modulate earthquake activity in volcanic areas^4–11,43^, providing information on the dynamics of different phases of the volcanic unrest. In addition, the similarities between the cases of El Hierro, 2011–2013, and La Palma, 2021, support the consistency of the conceptual models proposed for El Hierro^4^ and in this work. This model has potential application to the Canary Islands archipelago and to other volcanic islands, but additional research is needed to confirm this.

As the seismic monitoring of active volcanoes is essential in the evaluation of activity levels and potential hazards, a further understanding of the impact that tidal forces have on the seismic activity of active volcanoes can help improve risk assessment in warning systems, particularly in ocean island volcanoes where the influence of ocean tides may be greater than the effect of body tides.

## Methods

The methods we use are described in detail by Miguelsanz et al.^4^. What follows is a summary.

### Tidal stress

In order to obtain the tidal stress at the earthquake locations, it is necessary to consider both the solid earth and the ocean tides. In addition, the calculation must include the depth of the events^4^. Strains derived from the solid tides at depths less than 25 km are not significantly different from surface strains but strains due to the ocean loading component may change significantly^44^ between the surface and a depth of 25 km.

Our method uses the software package SPOTL^45^ for the calculation of tidal strains. Body tides are calculated on an elastic Earth following Munk and Cartwright^46^. The ocean tidal model we have used is osu.tpxo72atlas^45^, which is a hydrodynamic model assimilating altimetry data from TOPEX/Poseidon and Jason. It combines three basin-wide solutions (Atlantic Ocean 2011-atlas, Pacific Ocean 2011-atlas and Indian Ocean 2011-atlas), each one of which also incorporates a set of high-resolution local models. Tidal waves considered in the study are Mm, Mf, Q1, O1, P1, K1, N2, M2, S2, K2. The original SPOTL codes were modified to use depth-dependent Green functions in the calculations^4^, following the methodology described in Royer et al.^47^ for the modelling of tidal strains at depth. We added the load tides to the body tides to obtain the total tidal strain. Then tidal stresses are derived from tidal strains following Hooke’s law for a three-dimensional isotropic body^48^, with Poisson's ratio ν = 0.25 and rigidity μ = 30 GPa. We consider positive stress as extension.

### Tidal stress phase angle

Our method uses a modified version of the codes of the software package SPOTL^45^ for the calculation of tidal longitudinal strains, then implements Hooke’s law to derive the tidal stress components. A golden section search routine^49^ is used to estimate the extreme tidal stress values closest to the time of occurrence of the event. Finally, we assign a ‘phase angle’ *ϕ* to the earthquake by linear interpolation, supposing the time distance between two tidal peaks defines a complete cycle of 360° (see Fig. 3).

The Schuster test was used to determine if there is a non-random distribution of the calculated tidal phases^3^. In this test, each *i*th earthquake is assigned a unit length vector in the direction of the phase angle *ϕ*_*i*_. The modulus *R* of the vector sum **R** over the total number of earthquakes *N*_*tot*_ is given by

2 $${R}^{2}={\left({\sum }_{1}^{{N}_{tot}}\mathit{cos}{\phi }_{i}\right)}^{2}+{\left({\sum }_{1}^{{N}_{tot}}\mathit{Csin}{\phi }_{i}\right)}^{2},$$

where *C* is a factor correcting local asymmetries in tides^50^.

Then, the probability *P*_*s*_ that the phase distribution to be non-random is estimated by

3 $$P_{s} = \exp \left( {\frac{{ - R^{2} }}{{N_{tot} }}} \right)\;.$$

*P*_*s*_ represents the significance level with which to reject the null hypothesis that the earthquake phase angles show a totally random distribution. Small values of *P*_*s*_ (< 0.05) indicate the existence of a high correlation between tidal stresses and the occurrence of earthquakes.

The American Statistical Association (ASA) expressed some concerns regarding the hypothesis testing based on *p*-values, stating that these practices are prone to generate false positives^51^. Bayarri et al.^40^ propose a simple alternative to the use of *p*-values in testing null hypothesis, based on the Bayes factor *B*, which is defined as

4 $$B=\frac{average\,\, likelihood\,\, of\,\, the\,\, observed\,\, data \,\,under\,\, the\,\, alternative \,\,hypothesis}{likelihood \,\,of\,\, the\,\, observed\,\, data\,\, under\,\, the \,\,null \,\,hypothesis}.$$

Although *B* may be difficult to compute, it can be approximated by an upper bound $\overline{B }$ which is a function of the *p*-values obtained with the usual statistical tests used in testing hypothesis. In accordance with Bayarri et al.^40^, and considering the Schuster test as the appropriate one for our null hypothesis testing:

5 $$B\le \overline{B }= \frac{1}{-e{P}_{s}\mathrm{log}{P}_{s}}.$$

Here we use the *p*-values *P*_*s*_ calculated by means of the Schuster test to derive this upper bound $\overline{B }$ of the Bayes factor *B*. Again, according to Bayarri et al.^40^, the standard significant threshold for rejecting the null hypothesis when using this Bayesian alternative is $\overline{B }$ > 16.

### Permutation test

Because this study includes highly clustered seismicity, a Monte Carlo permutation test^41,52^ was performed to assess the significance of the probability value *P*_*s*_ and the upper bound $\overline{B }$ of the Bayes factor. For each one of the earthquake sets in our study, we ordered the events according to their phase angle *ϕ*, and we divided them into segments (phase angle ranges) of equal length. Different segment lengths were tested (see Table 3). Then, a large number *NSIM* of simulations (e.g. 10,000 simulations) were performed for each set with the segments in random order, obtaining a probability value *P*_s_* from the Schuster test for each simulation. Finally, for each phase we count the number *NSIG* of random simulations in which the *P*_s_* value obtained is lower than the *P*_*s*_ value of the original distribution of events. The ratio

6 $${MC}_{sl}=\frac{(NSIG+1)}{(NSIM+1)} ,$$

provides the significance level of our test^38^.

## Supplementary Information

Supplementary Information.

## Data Availability

Earthquake data catalogue used, for the period 2017/10/07–2021/12/31, can be obtained from Instituto Geográfico Nacional web site (https://www.ign.es/web/ign/portal/sis-area-sismicidad). The remainder of the data are available in the repository Zenodo.org (https://zenodo.org/record/7067330#.YxzZGXZBw2w). Also corresponding author (José Fernández, jft@mat.ucm.es) can be contacted to request the data from this study.
