# Supplementary material for: Tidal modulation of the seismic activity related to the 2021 La Palma volcanic eruption
Source: Sci Rep. 2023 Apr 20;13:6485. doi: 10.1038/s41598-023-33691-1 (PMC10119113; doi:10.1038/s41598-023-33691-1)
Supplement: Supplementary file 1 — Supplementary Information. [file 41598_2023_33691_MOESM1_ESM.pdf]

Supplementary Information for

Tidal modulation of the seismic activity related to the 2021 La Palma volcanic eruption

Luis Miguelsanz[1], José Fernández[1,*], Juan F. Prieto[2], and Kristy F. Tiampo[3]

[1]Institute of Geosciences (IGEO), CSIC-UCM, C/ Dr. Severo Ochoa, 7. 28040-Madrid, Spain.

[2]E.T.S. de Ingenieros en Topografía, Geodesia y Cartografía, Universidad Politécnica de Madrid, 28031-Madrid, Spain

[3]Cooperative Institute for Research in Environmental Sciences (CIRES), University of Colorado Boulder, Boulder, CO, USA

[*]Corresponding author: José Fernández (jft@mat.ucm.es)

This file includes:

Supplementary Figures. 1 to 15
Supplementary Tables 1 to 4

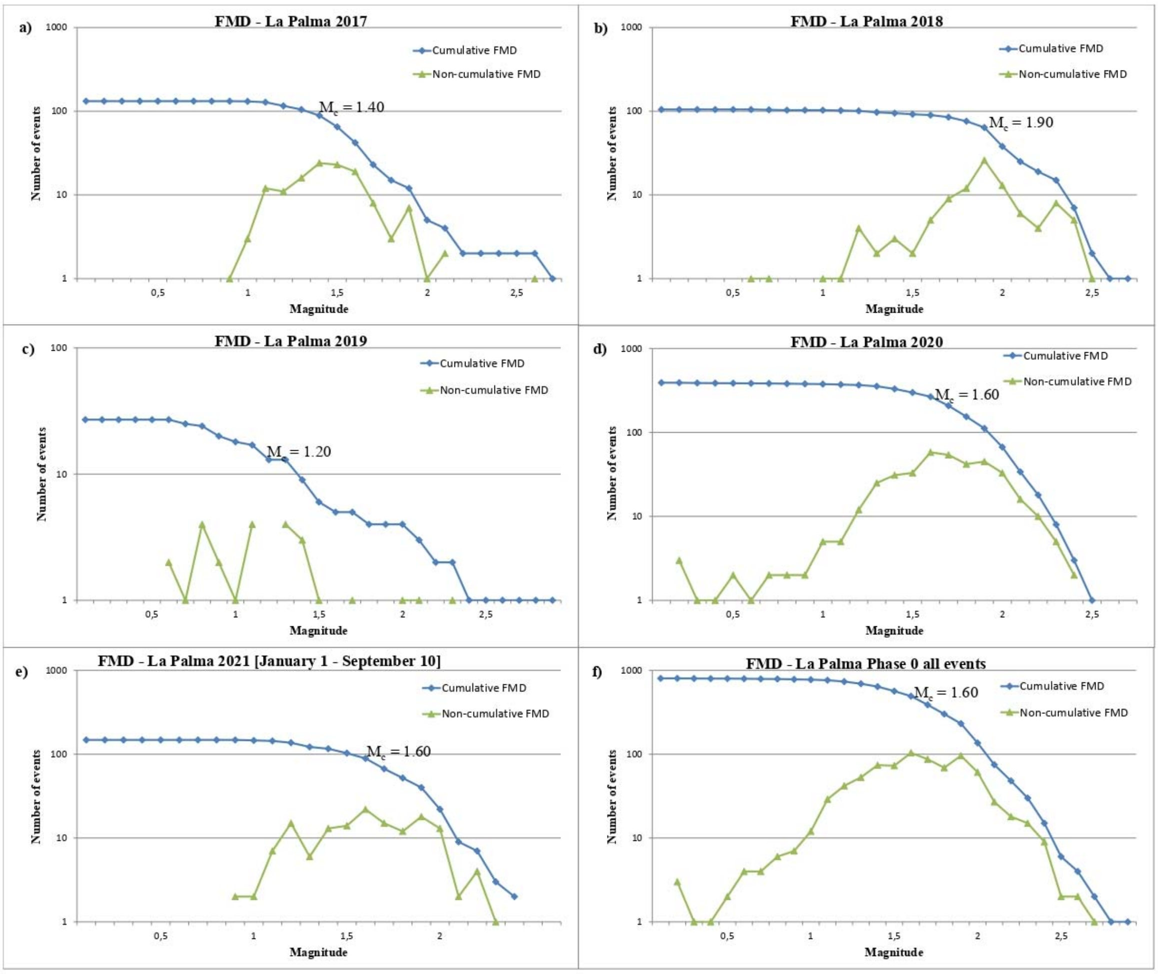

Supplementary Figure 1. Evolution of the frequency magnitude distribution (FMD) and the magnitude of completeness M_c of earthquakes in *Phase 0*, throughout the different years comprising that time period: a) 2017; b) 2018; c) 2019; d) 2020; e) 2021 (until the end of *Phase 0* on September 10th; f) all events in the whole *Phase 0* time period. Blue line is the cumulative FMD, whereas green line is the non-cumulative FMD.

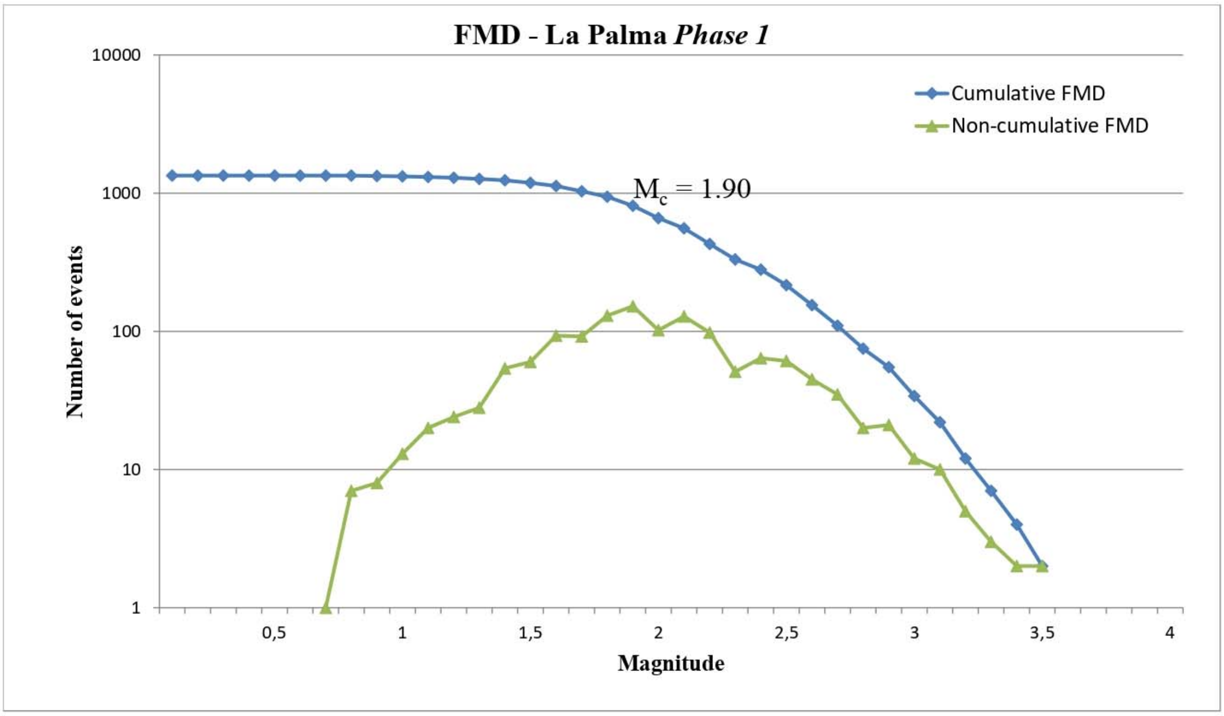

Supplementary Figure 2. Frequency magnitude distribution (FMD) and magnitude of completeness M_c of earthquakes in *Phase 1*. Blue line is the cumulative FMD; green line is the non-cumulative FMD.

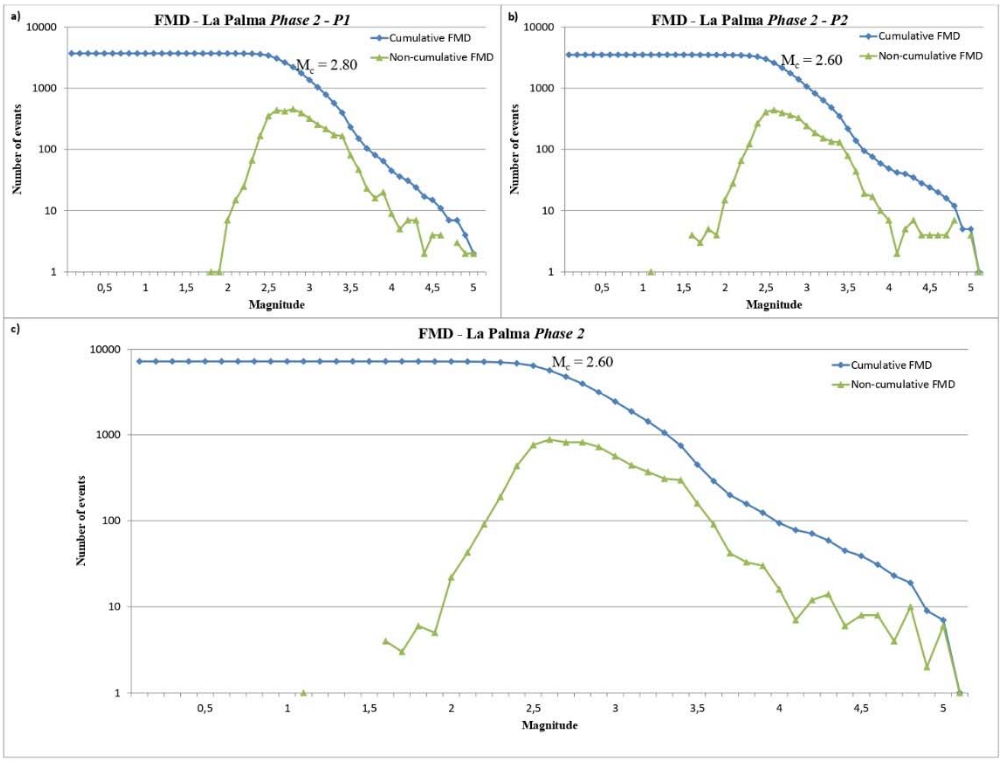

Supplementary Figure 3. Frequency magnitude distribution (FMD) and magnitude of completeness M_c of earthquakes in: a) *Phase 2 – P1* [2021/09/20 - 2021/11/01]; b) *Phase 2 – P2* [2021/11/02 - 2021/12/13]; c) *Phase 2* (complete co-eruptive period). Blue line is the cumulative FMD; green line is the non-cumulative FMD.

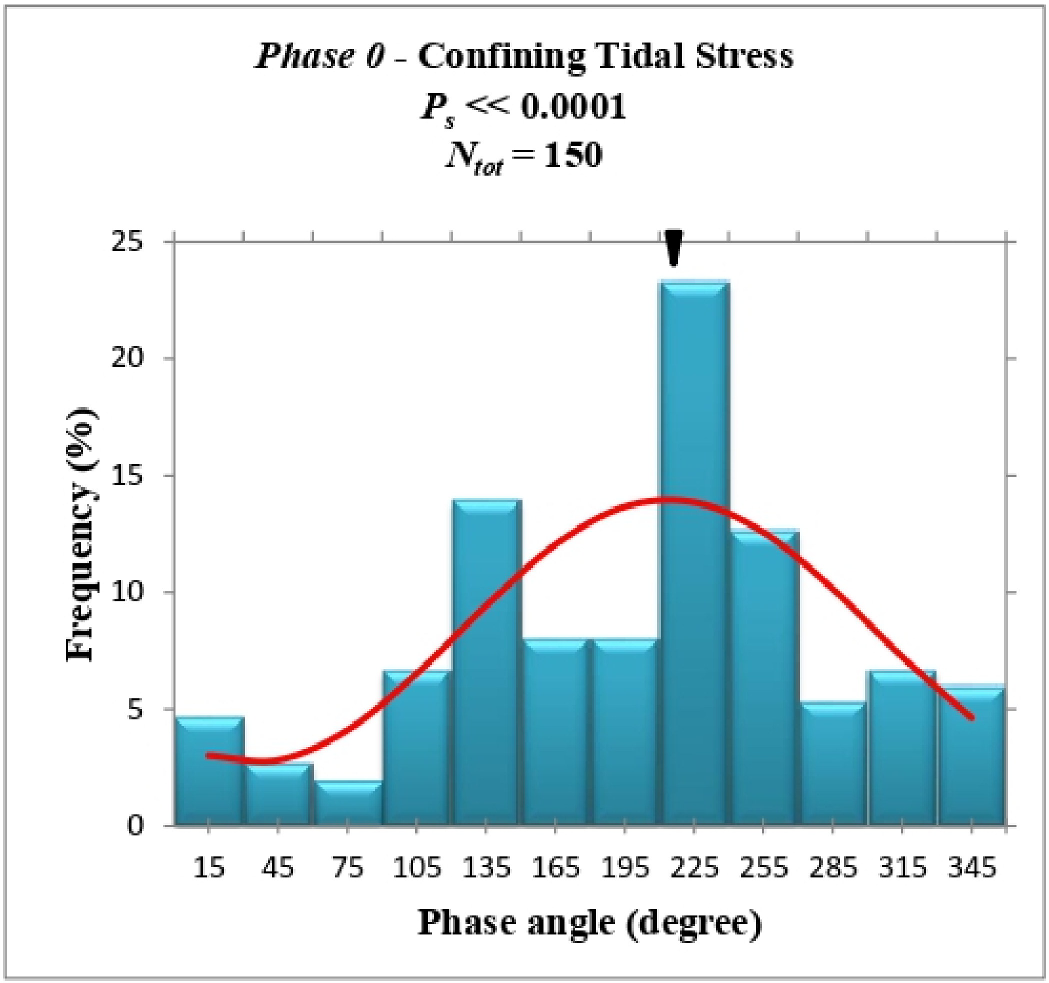

Supplementary Figure 4. Histogram of the frequency distribution of tidal phase angles for all events in *Phase 0* ($M \geq M_c$).. N_{tot} is the number of events. The curve represents a sinusoidal function fitted to the frequency distribution. The peak of the fitted curve is indicated by a triangle.

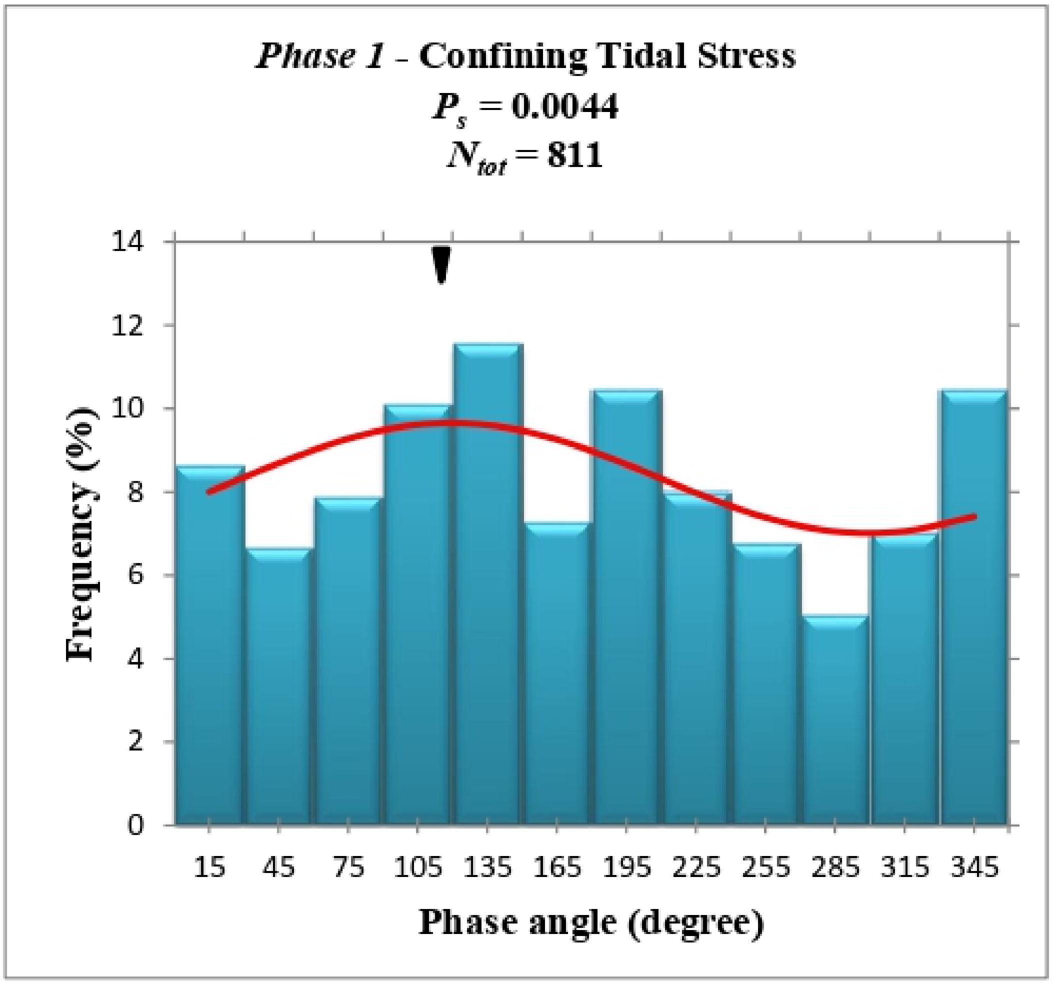

Supplementary Figure 5. Histogram of the frequency distribution of tidal stress phase angles for all events in *Phase 1* ($M \geq M_c$). N_{tot} is the number of events. The curve represents a sinusoidal function fitted to the frequency distribution. The peak of the fitted curve is indicated by a triangle.

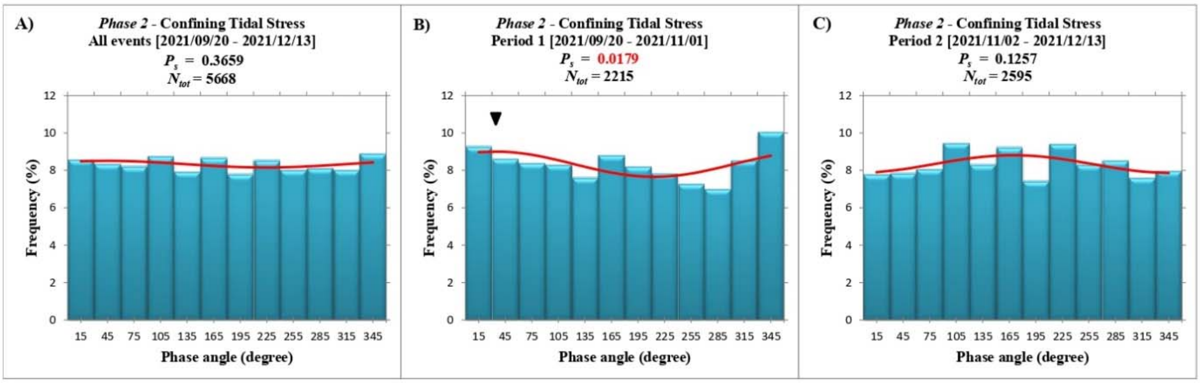

Supplementary Figure 6. Histogram of the frequency distribution of tidal stress phase angles for all events in *Phase 2* ($M \geq M_c$): A) All events until the end of eruption; B) Period 1 [2021/09/20 - 2021/11/01]; C) Period 2 [2021/11/02 - 2021/12/13]. N_{tot} is the number of events. The curve represents a sinusoidal function fitted to the frequency distribution. In those cases where a statistically significant correlation appears, a triangle marks the peak of the fitted curve, and the value of the statistical P_s is colored in red.

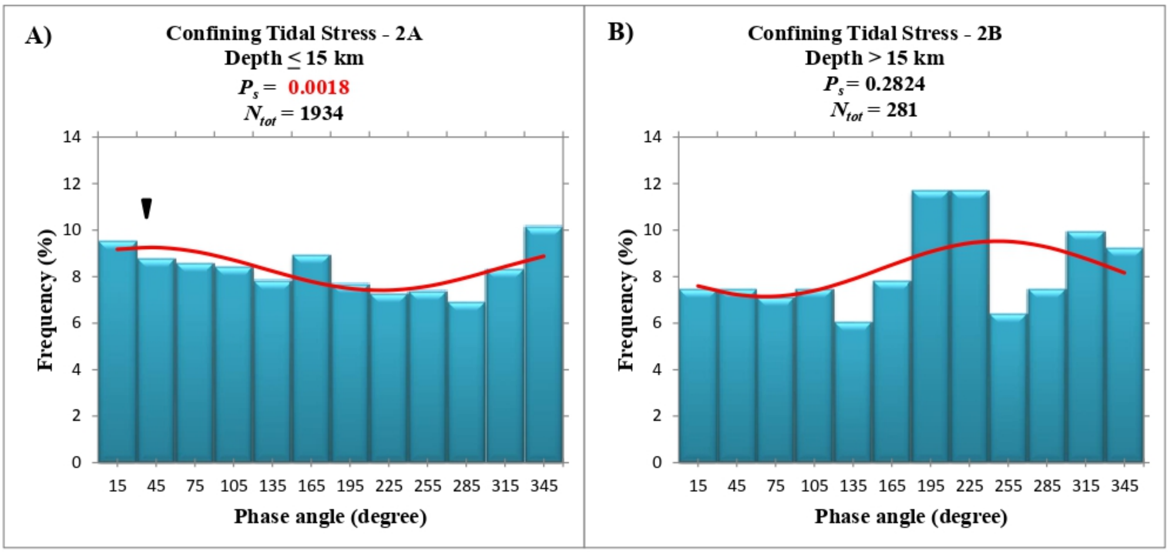

Supplementary Figure 7. Histograms of the frequency distribution of tidal stress phase angles for all events in: A) subset *2A*; B) subset *2B*. N_{tot} is the number of events. The curve represents a sinusoidal function fitted to the frequency distribution. In those cases where a statistically significant correlation appears, a triangle marks the peak of the fitted curve, and the value of the statistical P_s is colored in red.

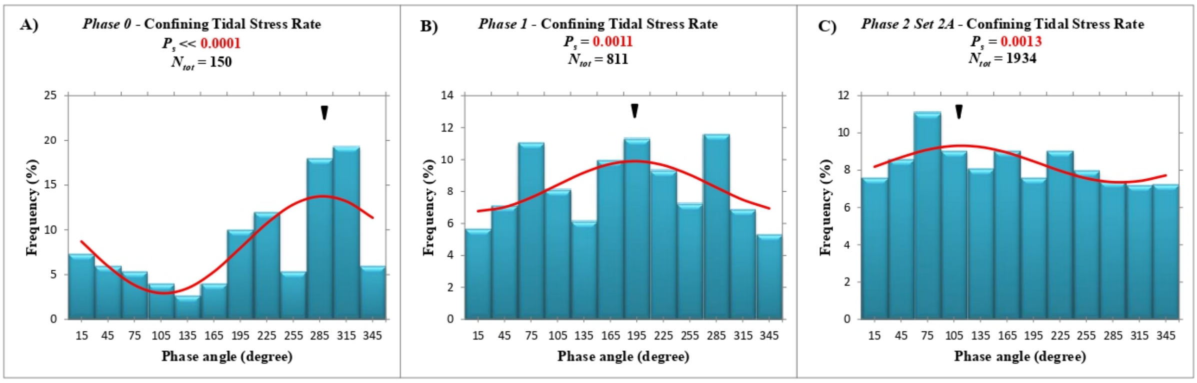

Supplementary Figure 8. Histograms of the frequency distribution of tidal stress rates phase angles for all events with $M \geq M_c$ in: A) *Phase 0*; B) *Phase 1*; C) *Phase 2 – Set 2A*. N_{tot} is the number of events. The curve represents a sinusoidal function fitted to the frequency distribution. In those cases where a statistically significant correlation appears, a triangle marks the peak of the fitted curve, and the value of P_s is colored in red.

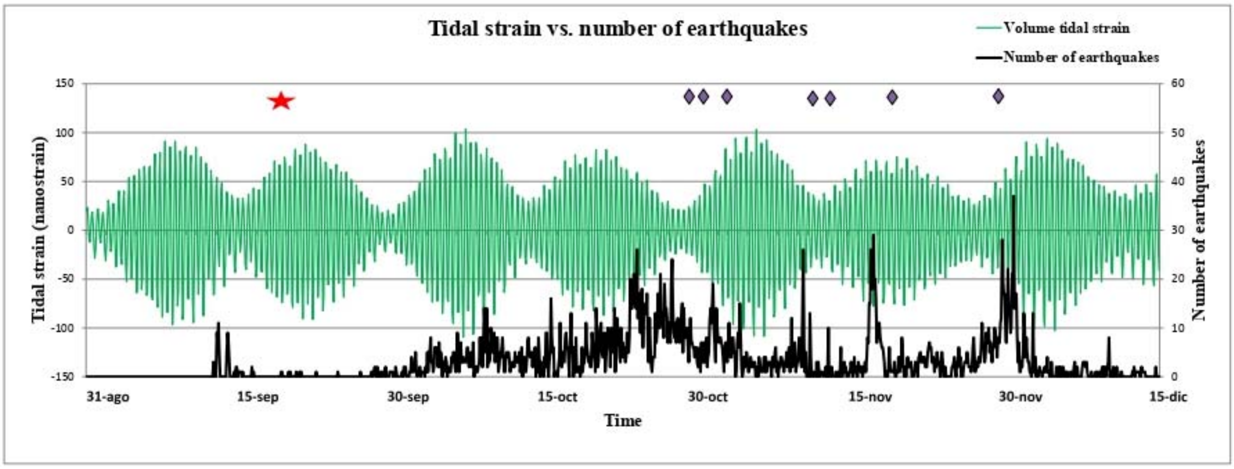

Figure 9. Green line shows tidal volume strain in La Palma during the period 2021/08/31 – 2021/12/15. Black line represents the number of earthquakes with magnitude $M \geq 2.8$ recorded every two hours during the same period. The purple diamonds mark the occurrence of the earthquakes with magnitude $M \geq 5$. The red star represents the time of the beginning of the eruption (September 19, 2021).

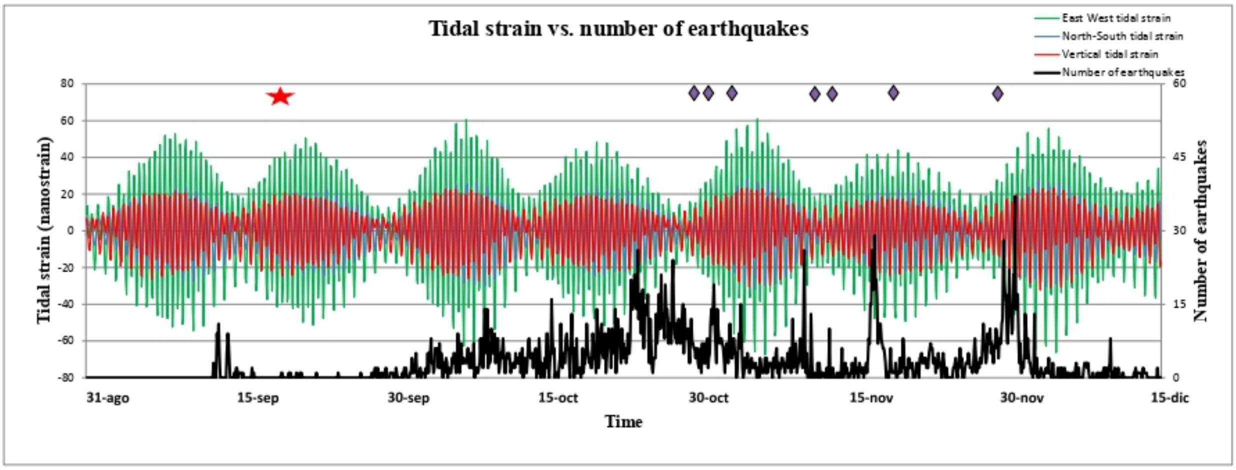

Figure 10. Tidal strain components (green line: east-west; blue line: north-south; red line: vertical) in La Palma during the period 2021/08/31 – 2021/12/15. Black line represents the number of earthquakes with magnitude $M \geq 2.8$ recorded every two hours during the same period. The purple diamonds mark the occurrence of the earthquakes with magnitude $M \geq 5$. The red star represents the time of the beginning of the eruption (September 19, 2021).

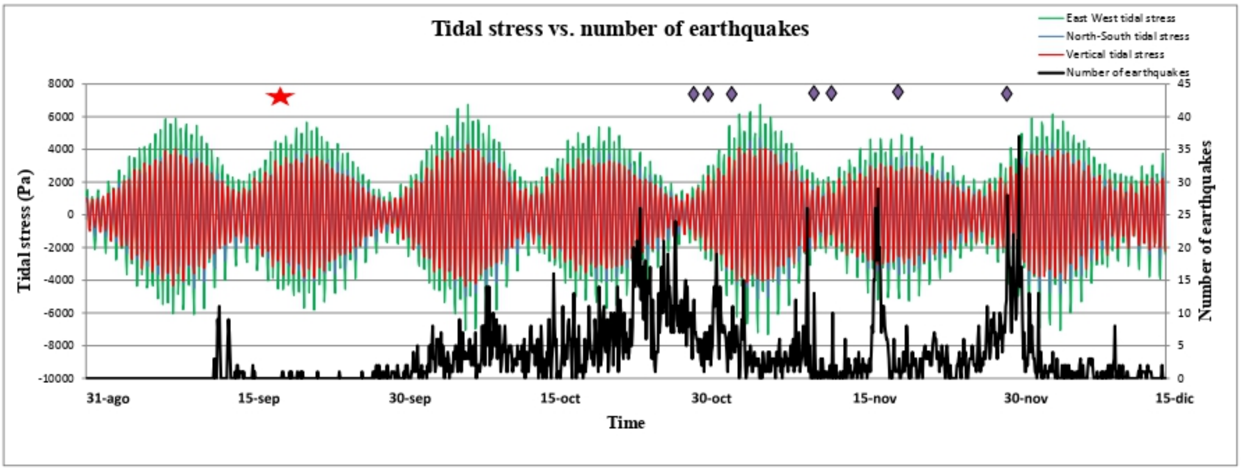

Figure 11. Tidal stress components (green line: east-west; blue line: north-south; red line: vertical) in La Palma during the period 2021/08/31 – 2021/12/15. Black line represents the number of earthquakes with magnitude $M \geq 2.8$ recorded every two hours during the same period. The purple diamonds mark the occurrence of the earthquakes with magnitude $M \geq 5$. The red star represents the time of the beginning of the eruption (September 19, 2021).

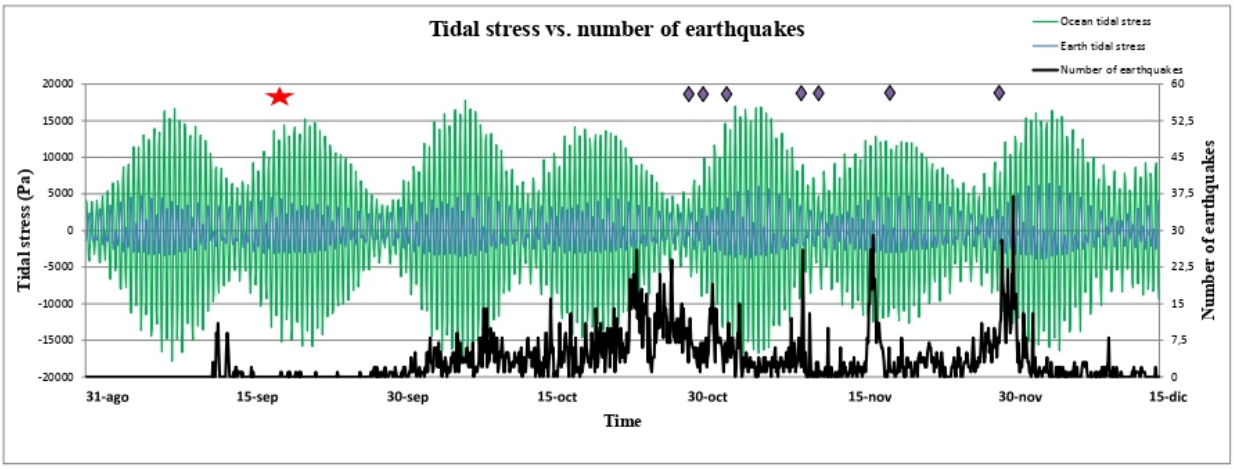

Figure 12. Ocean tidal stress (green line) and Earth tidal stress (blue line) in La Palma during the period 2021/08/31 – 2021/12/15. Black line represents the number of earthquakes with magnitude $M \geq 2.8$ recorded every two hours during the same period. The purple diamonds mark the occurrence of the earthquakes with magnitude $M \geq 5$. The red star represents the time of the beginning of the eruption (September 19, 2021).

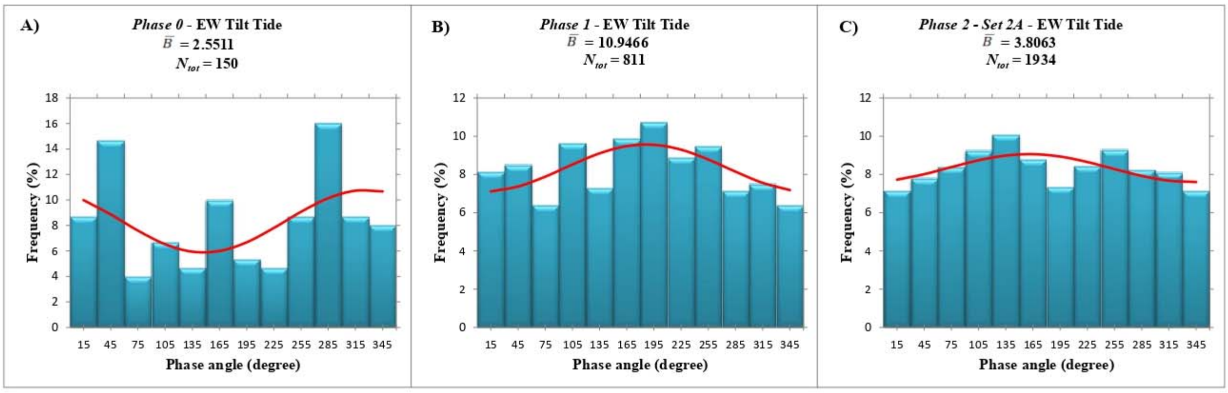

Supplementary Figure 13. Histograms of the frequency distribution of EW tidal tilt (ocean tides only) for all events with $M \geq M_c$ in: A) *Phase 0*; B) *Phase 1*; C) *Phase 2 – Set 2A*. $\overline{B}$ is the upper bound of the Bayes factor calculated as a function of the *p*-value, P_s, as discussed in the Methods section. N_{tot} is the number of events. The curve represents a sinusoidal function fitted to the frequency distribution.

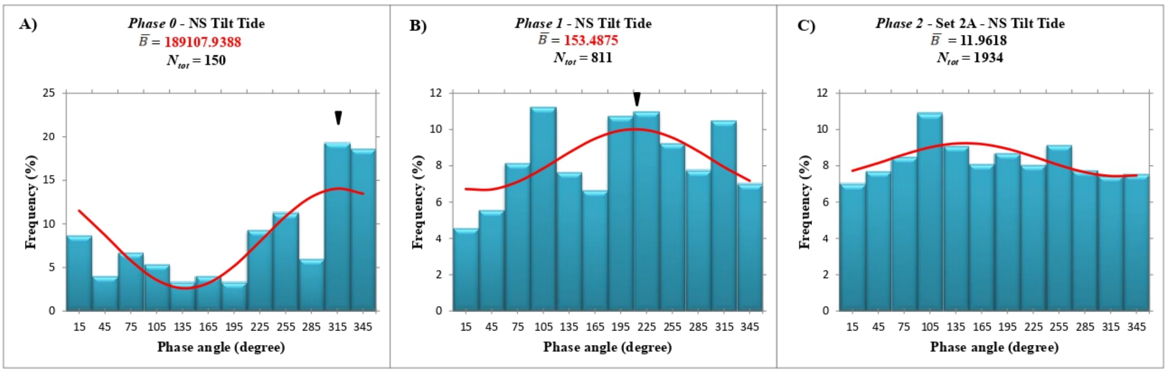

Supplementary Figure 14. Histograms of the frequency distribution of north-south tidal tilt (ocean tides only) for all events with M ≥ 2 in: A) *Phase 0*; B) *Phase 1*; C) *Phase 2 – Set 2A*. $\overline{B}$ is the upper bound of the Bayes factor calculated as a function of the *p*-value, P_s, as discussed in the Methods section. N_{tot} is the number of events. The curve represents a sinusoidal function fitted to the frequency distribution. In those cases where a statistically significant correlation appears, a triangle marks the peak of the fitted curve, and the value of the statistical $\overline{B}$ is colored in red.

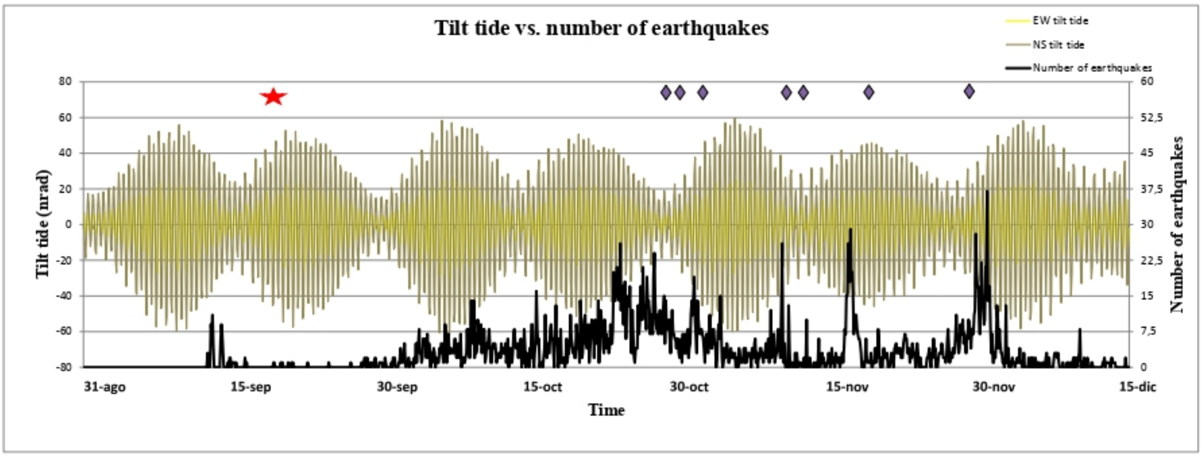

Figure 15. East-west tilt tide (yellow line) and north-south tilt tide (cinnamon line) in La Palma during the period 2021/08/31 – 2021/12/15. Black line represents the number of earthquakes with magnitude $M \geq 2.8$ recorded every two hours during the same period. The purple diamonds mark the occurrence of the earthquakes with magnitude $M \geq 5$. The red star represents the time of the beginning of the eruption (September 19, 2021).

Supplementary Table 1. Ratios N_n /N_{tot} for different values of the magnitude of completeness M_c considered for the events in *Phase 1*[a]

M_c	N_{tot}	N_d	N_n	Ratio (N_n /N_{tot})
1,7	1034	378	656	0,634429400
1,8	941	348	593	0,630180659
1,9	811	305	506	0,623921085
2	659	241	418	0,634294385
2,1	557	198	359	0,644524237
2,2	429	129	300	0,699300699

[a] M_c is the magnitude of completeness of the catalog, N_{tot} is the total number of events with magnitude $M \geq M_c$. N_d is the number of events with $M \geq M_c$ recorded in daily hours (between 6:00 and 18:00) and N_n is the number of events with $M \geq M_c$ recorded in nightly hours.

Supplementary Table 2. List of seismic stations in La Palma, with start and termination dates (https://www.ign.es/web/ign/portal/vlc-estaciones-red/-/estaciones-canarias/setTabIGN#)

Station	**Start Date**	**Termination Date**
TBT	1974/11/01	--
EHIG	2002/01/18	--
CJED	2017/05/24	--
CPUN	2017/05/24	--
CTEN	2017/05/25	--
CENR	2017/10/10	--
CROM	2017/10/10	--
CVIE	2017/10/10	2021/04/08
CFLP	2017/11/06	2019/03/20
CGOR	2017/11/07	--
CMIR	2017/11/08	--
CLLA	2017/11/09	2019/01/13
CMAZ	2017/11/10	2018/01/17
CBRE	2018/04/03	--
CALD	2021/04/08	
CAVE	2021/09/21	

Supplementary Table 3. Summary of the correlations between EW tidal tilt (ocean tides only) and origin time of the events[a]

**Phase**	**Period**	**Number of events ($M \geq M_c$)**	**P_s value**	**$\overline{B}$**
Phase 0	2017/10/07 - 2021/09/10	150	**0.0472**	2.5511
Phase 1	2021/09/11 - 2021/09/19	811	**0.0067**	10.9466
Phase 2 – P1	2021/09/20 - 2021/11/01	2215	0.1154	1.4762
Phase 2 – P2	2021/11/02 - 2021/12/13	2595	0.2478	1.0641
Phase 2 – Set 2A	2021/09/20 - 2021/11/01	1934	**0.0267**	3.8063

[a]M_c is the magnitude of completeness, which is different in every set or subset of events. See Section 3. P_s is the probability that the phase distribution to be random, according to Schuster's test. $\overline{B}$ is the upper bound of the Bayes factor calculated as a function of the p-value P_s, as told in the Methods section. Occurrences where $P_s < 0.05$ or $\overline{B} > 16$ are in bold. Note that *Phase 2* has been divided in two periods: *P1* [2021/09/20 - 2021/11/01] and *P2* [2021/11/02 - 2021/12/13].

Supplementary Table 4. Summary of the correlations between NS tidal tilt (ocean tides only) and origin time of the events[a]

Phase	Period	Number of events (**$M \geq M_c$**)	**P_s value**	**$\overline{B}$**
Phase 0	2017/10/07 - 2021/09/10	150	**<< 0.0001**	**189107.9388**
Phase 1	2021/09/11 - 2021/09/19	811	**0.0003**	**153.4875**
Phase 2 – P1	2021/09/20 - 2021/11/01	2215	**0.0292**	3.5696
Phase 2 – P2	2021/11/02 - 2021/12/13	2595	0.0997	1.6006
Phase 2 – Set 2A	2021/09/20 - 2021/11/01	1934	**0.0060**	11.9618

[a]M_c is the magnitude of completeness, which is different in every set or subset of events. See Section 3. P_s is the probability that the phase distribution to be random, according to Schuster's test. $\overline{B}$ is the upper bound of the Bayes factor calculated as a function of the *p*-value P_s, as told in the Methods section. Occurrences where $P_s < 0.05$ or $\overline{B} > 16$ are in bold. Note that *Phase 2* has been divided in two periods: *P1* [2021/09/20 - 2021/11/01] and *P2* [2021/11/02 - 2021/12/13].
